# Using global transcription machinery engineering (gTME) to improve ethanol tolerance of *Zymomonas mobilis*

**DOI:** 10.1186/s12934-015-0398-y

**Published:** 2016-01-13

**Authors:** Furong Tan, Bo Wu, Lichun Dai, Han Qin, Zongxia Shui, Jingli Wang, Qili Zhu, Guoquan Hu, Mingxiong He

**Affiliations:** Biogas Institute of Ministry of Agriculture, Biomass Energy Technology Research Centre, Section 4-13, Renmin Nanlu, Chengdu, 610041 China; Key Laboratory of Development and Application of Rural Renewable Energy, Ministry of Agriculture, Chengdu, 610041 China

**Keywords:** Ethanol tolerance, *Zymomonas mobilis*, Random mutagenesis, Global transcription machinery engineering (gTME), Error-prone PCR, σ^70^

## Abstract

**Background:**

With the increasing global crude oil crisis and resulting environmental concerns, the production of biofuels from renewable resources has become increasingly important. One of the major challenges faced during the process of biofuel production is the low tolerance of the microbial host towards increasing biofuel concentrations.

**Results:**

Here, we demonstrate that the ethanol tolerance of *Zymomonas mobilis* can be greatly enhanced through the random mutagenesis of global transcription factor RpoD protein, (σ^70^). Using an enrichment screening, four mutants with elevated ethanol tolerance were isolated from error-prone PCR libraries. All mutants showed significant growth improvement in the presence of ethanol stress when compared to the control strain. After an ethanol (9 %) stress exposure lasting 22 h, the rate of glucose consumption was approximately 1.77, 1.78 and 1.39 g L^−1^ h^−1^ in the best ethanol-tolerant strain ZM4-mrpoD4, its rebuilt mutant strain ZM4-imrpoD and the control strain, respectively. Our results indicated that both ZM4-mrpoD4 and ZM4-imrpoD consumed glucose at a faster rate after the initial 9 % (v/v) ethanol stress, as nearly 0.64 % of the initial glucose remained after 54 h incubation versus approximately 5.43 % for the control strain. At 9 % ethanol stress, the net ethanol productions by ZM4-mrpoD4 and ZM4-imrpoD during the 30–54 h were 13.0–14.1 g/l versus only 6.6–7.7 g/l for the control strain. The pyruvate decarboxylase activity of ZM4-mrpoD4 was 62.23 and 68.42 U/g at 24 and 48 h, respectively, which were 2.6 and 1.6 times higher than the control strain. After 24 and 48 h of 9 % ethanol stress, the alcohol dehydrogenase activities of ZM4-mrpoD4 were also augmented, showing an approximate 1.4 and 1.3 times increase, respectively, when compared to the control strain. Subsequent quantitative real-time PCR analysis under these stress conditions revealed that the relative expression of *pdc* in cultured (6 and 24 h) ZM4-mrpoD4 increased by 9.0- and 12.7-fold when compared to control strain.

**Conclusions:**

Collectively, these results demonstrate that the RpoD mutation can enhance ethanol tolerance in *Z. mobilis*. Our results also suggested that RpoD may play an important role in resisting high ethanol concentration in *Z. mobilis* and manipulating RpoD via global transcription machinery engineering (gTME) can provide an alternative and useful approach for strain improvement for complex phenotypes.

## Background

With the increasing global crude oil crisis and resulting environmental concerns, the production of biofuels from renewable resources has become increasingly important [1]. To this end, bioethanol production has seen a sharp escalation over the past decades. In general, bioethanol can be produced by fermenting biological resources (e.g. energy-rich crops or lignocellulosic biomass) using a variety of potential microbes such as *Saccharomyces cerevisiae*, *Escherichia coli*, *Klebsiella oxytoca*, and *Zymomonas mobilis* [2]. Of these, *Z. mobilis*, a Gram-negative facultative anaerobic bacterium, has attracted considerable interest. Critically, it has a unique metabolism and ability to produce ethanol and/or other valuable chemicals from simple sugars via its unique Entner–Doudoroff (ED) pathway [3–6].

However, various environmental stressors can both adversely affect the microorganism growth of *Z. mobiles* and its ability to produce ethanol during fermentation. For instance, high ethanol concentrations, osmotic pressure, and oxidative stresses are all major stress that can impede the specific growth rate and viability of *Z. mobilis* cells as well as its ethanol production [7–9]. To better understand and address these limitations, it is essential to obtain mutant *Z. mobilis* strains that have improved stress tolerance [7, 10–14].

Past work has established that multi-gene regulation involving carbohydrate metabolism, cell membrane biogenesis, respiratory chain, DNA replication and recombination, transcriptional regulation, and some universal stress responses culminates in the stress tolerance of *Z. mobilis* [15–17]. Similarly, the genes associated with ethanol tolerance in yeast were also found to be linked to a broad range of different functional categories and biological functions [18, 19]. Recently, Henderson and Block (2014) also revealed that in *S. cerevisiae*, the lipid composition of the cellular membrane plays an important role in its response to ethanol stress [20]. Although many studies have been carried out to better understand the molecular basis of ethanol stress and tolerance in *S. cerevisiae,* it is still a challenging and difficult task to construct a wide enough variety of strains capable of responding to various stresses. The recent development of the global transcriptional engineering has attracted much attention in the field of strain engineering as a possible solution to this problem, particularly for those working on stress tolerance. Several transcription factors, including zinc finger-containing artificial transcription factor [21–23], sigma factor [24, 25], Spt15 [26], H-NS [27], Hha [28], and cAMP [29, 30], have been modified via global transcriptional engineering for improved strain tolerance and better control of biofilm formation. With this methodological development, a new route for identifying mutant transcription factor that can tolerate various inhibitors has been established. However, little work using global transcriptional engineering has focused on genetically improving the stress tolerance of *Z. mobilis*.

Since the RNA polymerase sigma subunit (σ factor) is known as a fundamental in promoter recognition and transcriptional initiation at the correct site, mutation of σ factor might alter the promoter preferences of RNA polymerase. In turn, this could affect transcriptional levels, thus modulating the transcriptome on a global level. We thus sought to improve the ethanol tolerance of *Z. mobilis* ZM4 by engineering its *rpoD* gene, which encodes the main sigma factor, σ^70^. The *rpoD* gene was subjected to error-prone PCR and cloned into a low-copy expression vector, pBBR1MCS-tet. Recombinant plasmids were then transformed into *Z. mobilis* ZM4 and random mutagenesis libraries were subjected to selection pressure using ethanol as a stress. Using this method, four error-prone PCR mutants with enhanced ethanol resistance were identified, all of which showed increased ethanol tolerance when compared to wild type. The mutant demonstrating the highest resistance, ZM4-mrpoD4, was subjected to further evaluation of its glucose utilization and key enzymatic activity. Finally, quantitative real-time PCR analysis was performed to detect the expression levels of several genes related to *Z. mobilis* metabolic pathways.

## Methods

### Materials

*E. coli* DH5α was cultured in LB medium and used as the host organism for all common transformations. Plasmid pBBR1MCS-tet was derived from pBBR1MCS [31]. Restriction enzymes were purchased from Fermentas (Burlington, Canada). E.Z.N.A.^®^ Gel Extraction Kit and E.Z.N.A. Plasmid Mini Kit I were obtained from Omega Bio-Tek (Norcross, GA, USA). T4 DNA ligase was obtained from Thermo Scientific (Ipswich, MA, USA) and was used for ligations. GeneMorph^®^ II Random Mutagenesis Kit was obtained from Stratagene (La Jolla, CA, USA). HotMaster Taq DNA polymerase was obtained from Tiangen Biotech (Beijing, China). The primers used in this study are summarized in Table 1.

**Table 1:** Primer sequences with restriction site underlined

Primer	Sequence
Ppdc KpnI F	5′-CGG GGTACCTTACGCTCATGATCGC-3′
Ppdc XhoI R	5′-CCG CTCGAG TGCTTACTCCATATAT-3′
1623 XhoI F	5′-CCG CTCGAG ATGGCAGAGACGACTACGG-3′
1623 XbaI R	5′-TGCTCTAGACTAGTGGTCGAGGAAGCTCC-3′
Tpdc XbaI F	5′-GACGGCTCTAGATAGTTTTTAAATAAACTTAGAGCTTAAG-3′
Tpdc SacI R	5′-GCCTCGAGCTCAATTTTATAGAAAAGAAAAACAAAGC-3′
ZMO1360 F	5′-TACAACCTCGTCCTTCTT-3′
ZMO1360 R	5′-CATAACCTTCTGCACTGA-3′
ZMO1596 F	5′-GGTATTAATTCTGCTGTT-3′
ZMO1596 R	5′-CGAAGTCTGAATTGTTAT-3′
16s F	5′-TCAACTATAGACCAGTAAGT-3′
16s R	5′-AGAACATAGAAGAGGTAAGT-3′

### Construction of random mutagenesis libraries

**Fig. 1:**
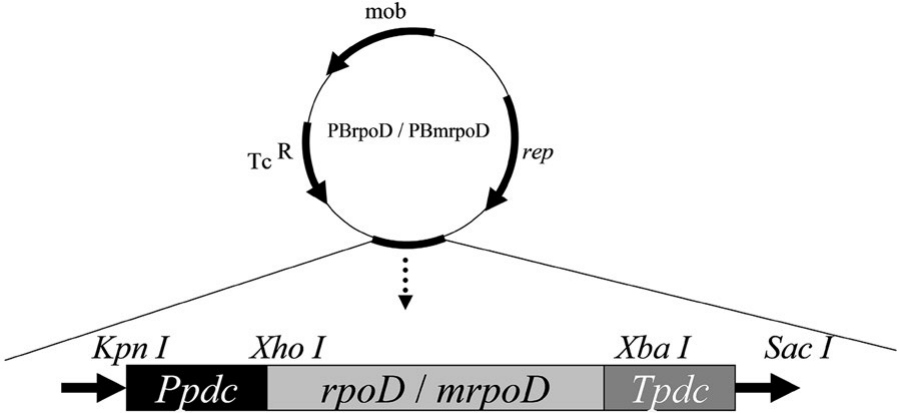
Schematic illustration of recombinant plasmids pBrpoD or pBmrpoD. Ppdc and Tpdc indicate the promoter and terminator of pyruvate decarboxylase, respectively

Error-prone PCR was performed using 180 ng of *rpoD*. Resulting PCR products were then subjected to the GeneMorph II Random Mutagenesis Kit (Stratagene) using various concentrations of initial template. This approach yielded low (0–4.5 mutations/kb), medium (4.5–9 mutations/kb), and/or high mutation (9–16 mutations/kb) rates as described in the manufacturer’s protocol. Following PCR, fragments were purified using E.Z.N.A.^®^ Gel Extraction Kit (Norcross, GA, USA) according to the manufacturer’s instructions, digested by *Xho* I and *Xba* I, and ligated into the corresponding restriction sites of pBBR1MCS-tet, which contained the pyruvate decarboxylase (PDC) promoter and terminator in order to generate either the recombinant plasmid PBmrpoD or PBrpoD (harboring the unmutated version of *rpoD* gene) (Fig. 1). Plasmids were then transformed into *Z. mobilis* ZM4 via electroporation, after which cells were plated on RM-agar plates containing 5 μg/ml of tetracycline for culturing 4–5 day and scraped off to create a liquid library.

### Phenotype selection

Transformants were incubated in 5 ml RM medium at 30 °C without shaking. One percent of the overnight cell culture was then inoculated into fresh RM supplemented with increasing initial ethanol concentration (7, 8, and 9 % (v/v), sequentially) for 24 h. After three rounds of selection, cells were spread onto RM-agar plates containing 5 μg/ml of tetracycline and the ethanol (9 %) stress. Individual colonies were randomly selected, plasmids extracted, and mutations verified using DNA sequencing. All mutant strains were compared to control strains *Z. mobilis* ZM4 and ZM4-rpoD, which harbored the recombinant plasmid PBrpoD as described above. By using controls, the influence of the plasmid and/or any interference between the plasmid and chromosomal copies of *rpoD* were neutralized.

### Cell growth profiling

To generate growth curves for both the mutant and control strains, cells were cultivated in a Bioscreen C system (Lab Systerms Helsinki, Finland) according to the manufacturer’s instructions. Briefly, 1:10 of the overnight seed (v/v) was inoculated into 1 ml fresh RM medium containing a range of initial ethanol concentrations [0, 6, 8 and 10 % (v/v)] with a similar initial OD_600_ value between 0.15–0.2. Cells were then added in triplicate into the wells of the Bioscreen plate with an end working volume of 300 μl/well. Temperature was controlled at 30 °C and the OD at 600 nm. Absorbance values of the cell suspensions were automatically read at regular intervals of 1 h over a 48 h time period. Before each measurement, cell cultures were automatically shaken for 60 s to homogenize the samples.

### Glucose utilization and ethanol analysis under ethanol stress condition

The mutant *rpoD* gene from the best ethanol-tolerant strain was cloned back to freshly prepared pBBR1MCS-tet plasmids described above, back-transformed into wild type strain ZM4 to proof that only this mutation is responsible for phenotype. Cells were grown in RM medium containing 20 g/l glucose at 30 °C to the mid-log phase. Ten ml of the culture was then transferred into 100 ml fresh RM medium (50 g/l glucose) containing 9 % (v/v) ethanol with a initial OD_600_ value of approximately 0.2. Cells were grown at 30 °C for 2–3 days. The OD_600_ was monitored by measuring the optical density at 600 nm with a UV765 spectrophotometer. Glucose and ethanol were measured using high performance liquid chromatography (HPLC) (Agilent Hi-plex H, 300 × 7.7 mm) with sulfuric acid (0.05 M) as the mobile phase, a flow rate of 0.6 mL/min, and a column temperature of 35 °C. The net ethanol production was calculated by the total ethanol minus the initial 9 % ethanol.

### Quantitative-PCR analysis

Total RNA was isolated using Trizol reagent and the resulting RNA samples were reverse-transcribed using the QuantScript RT Kit (TIANGEN, Beijing, China) as described in the manufacturer’s protocol. The expression of representative identified genes (*adhB* and *pdc*) from different treatments were quantified by quantitative real-time PCR (qPCR) using a BIO-RAD Real-Time PCR-iQ5 System. All optimized primers are shown in Table 1 and were designed using primer software to amplify approximately 100 bp from the 3′ end of the target genes. PCR conditions were 15 min at 94 °C, followed by 40 cycles of heating at 94 °C for 20 s and 50–55 °C for 30 s, and 72 °C for 20 s, and final extension at 72 °C for 5 min. PCR amplification was detected by SYBR Green (TIANGEN, Beijing, China). The ratios of the cycle threshold (Ct) values were determined from the included BIO-RAD iQ5 Optical System Software. The ΔΔCt method was chosen to analyze gene expression levels and standard curves for each primer were plotted to ensure similar amplification efficiency when compared with the reference gene. The *rrsA* gene, encoding the 16S RNA, served as an endogenous control to normalize for differences in total RNA quantity.

### Enzyme assay

Pyruvate decarboxylase (PDC) activity was determined by a previously described method [32] by monitoring the pyruvicacid-dependent oxidation of NADH with ADH as a coupling enzyme at pH6.5. The reaction was carried out at 25 °C in 50 mM sodium citrate buffer (pH6.5) containing 0.15 mM NADH, 5 mM MgCl_2_, 0.1 mM TPP, 5 mM pyruvate, and 10 μl (10 U) of ADH. The reaction was started by the addition of 10 μl of cell-free extract. The rate of NADH oxidation was measured at 340 nm.

Alcohol dehydrogenase (ADH) activity was assayed by measuring the alcohol-dependent reduction of NAD^+^ at pH 6.5. Cells were permeabilized using methods designed for enzymatic assays as previously described [33, 34]. Cell lysates (10–30 μl) were added for a final volume of 1 ml (333 mM ethanol, 8.3 mM NAD^+^ in 50 mM sodium phosphate buffer, pH 6.5). The production of NADH was assayed from the change in absorbance at 340 nm. One unit of PDC/ADH activity was defined as the generation of 1 μmol NAD^+^/NADH per minute under the conditions specified. Enzyme activities were reported as international units per milligram of total cell protein. Protein was measured by the Lowry method with bovine serum albumin as a standard.

## Results and discussion

### Isolation of ethanol-tolerant RpoD mutants

Recombinant plasmids PBmrpoD were transformed into competent *Z. mobilis* ZM4 and the subsequent mutagenesis libraries were tested in subcultures with repeated ethanol [7, 8 and 9 % (v/v)] administration to separate those successful mutants harboring enhanced ethanol tolerance. Cells were spread onto RM-agar plates containing 5 μg/ml of tetracycline and the initial ethanol (9 %) stress. Using this method, approximately several dozen strains were initially screened from the RM-agar plates. Of these, four ethanol-tolerant mutant strains (ZM4-mrpoD1, ZM4-mrpoD2, ZM4-mrpoD3 and ZM4-mrpoD4) that had markedly better cell growth under ethanol stress were selected for further analysis.

These four mutants were compared in growth performance between ZM4 and ZM4-rpoD. Initial studies of growth characteristics of strains in the presence of initial ethanol 7 % (v/v) indicated that both this control strain and an alternative control containing only a blank plasmid (no *rpoD* gene) had similar growth rates. As a result, we chose to use the strain ZM4-rpoD as the sole control strain for all further experiments presented here.

### RpoD mutant growth

**Fig. 2:**
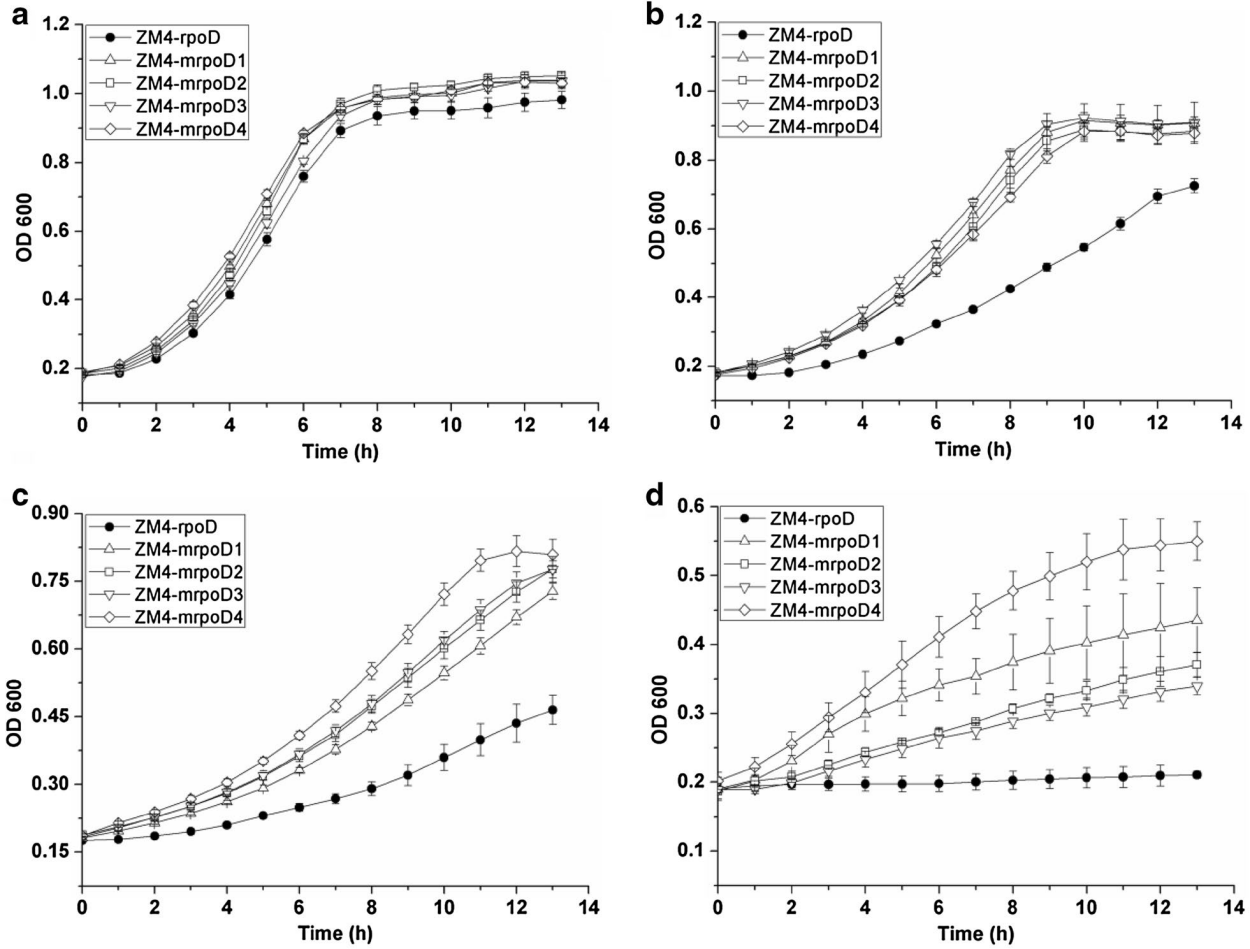
Growth of RpoD mutants and control strain ZM4-rpoD in RM medium. Control strain ZM4-rpoD contains the un-mutated version of *rpoD* gene, cells were grown in **a** 0 % ethanol, **b** 6 % ethanol, **c** 8 % ethanol and **d** 10 % (v/v) ethanol stress at 30 °C

We then sought to investigate the effects of ethanol stress on the growth of the RpoD mutant and control strains. The resulting growth curves are presented in Fig. 2. The ethanol tolerances of the four ethanol-tolerant mutants were investigated at different initial ethanol [6, 8 and 10 % (v/v)] concentrations by comparing their growth performance to that of the control. When cultured without initial ethanol addition, all mutants and control presented similar cell growth curve (Fig. 2). As the initial ethanol concentration increased in the culture medium, all mutants showed better growth than control, with mutant ZM4-mrpoD4 displaying the best ethanol tolerance of the four. As shown in Fig. 2, in the presence of 6 % ethanol stress, ZM4-mrpoD4 entered the plateau phase after 7–8 h, which was significantly ahead (3 h) of the control strain. When initial ethanol concentration reached 8 % (v/v), ZM4-mrpoD4 reached its highest cell density of 0.9 (OD_600_), while that of control strain was 0.4. When the initial ethanol concentration was increased to 10 % (v/v), all strains growth slowed, but mutant growth remained much faster than that of control.

### Effect of ethanol stress on glucose utilization and ethanol production

**Fig. 3:**
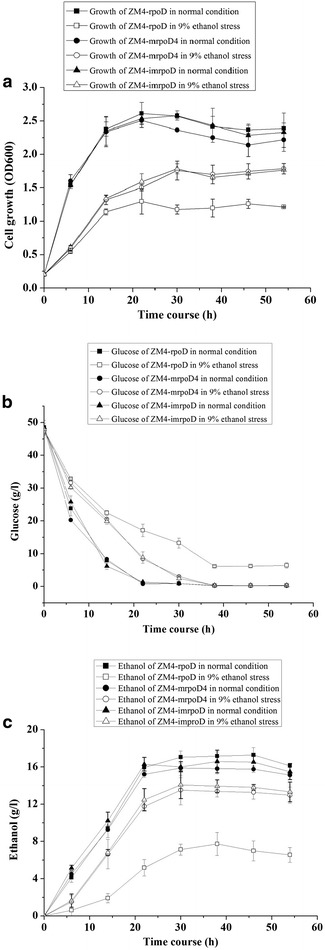
Effects of ethanol stress on growth, glucose utilization, and ethanol yield of mutant strains and control strain. Cells were grown in normal condition and 9 % ethanol stress, data are presented as the mean values of samples run in triplicate. **a** cell growth (OD_600_); **b** glucose concentration (g/l); **c** ethanol (g/l)

Since the mutant ZM4-mrpoD4 had demonstrated the best ethanol resistance among all four mutants, its mutant *rpoD* gene was cloned back to freshly prepared pBBR1MCS-tet plasmids and back-transformed into wild type strain ZM4 to create strain ZM4-imrpoD. During ethanol fermentation, ethanol stress may adversely affect the ability of the cell to perform efficient and consistent conversion of sugars to ethanol. Given this, we sought to examine the influence of the RpoD mutation on the fermentation ability of *Z. mobilis* (Fig. 3). From the Fig. 3, there were no differences between mutant strains (ZM4-mrpoD4 and ZM4-improD) and control strain ZM4-rpoD in terms of the growth, glucose utilization, and ethanol yield at normal condition. However, in the RM medium containing ethanol (9 %, v/v) stress, ZM4-mrpoD4 and ZM4-imrpoD reached their maximal cell density (OD_600_) at approximately 1.8 after the initial 30 h incubation. Comparatively, the control strain reached its highest cell density of approximately 1.2 (OD_600_) under the same condition. After 22 h of the ethanol (9 %) stress, the rate of glucose consumption was about 1.77, 1.78 and 1.39 g L^−1^ h^−1^ in ZM4-mrpoD4, ZM4-imrpoD and control strain, respectively. These data clearly indicated that ZM4-mrpoD4 and ZM4-imrpoD consumed glucose faster under ethanol stress conditions, as nearly 18 % of the initial glucose remaining after 22 h incubation, versus about 36 % for the control strain. When fermented for 54 h in the presence of ethanol (9 %, v/v), the initial glucose remained in the cultures of the control strain and mutants strains was approximately 5.43 and 0.64 %, respectively. We also measured the net ethanol productions of mutant strains and control strain under the process of fermentation at normal condition and 9 % ethanol stress. Our results indicated that the net ethanol production by ZM4-mrpoD4 and ZM4-imrpoD during the 30–54 h were 13.0–14.1 g/l versus only 6.6–7.7 g/l for the control strain, thus indicating that ZM4-mrpoD4 and ZM4-imrpoD can produce more ethanol than the control strain under the condition of 9 % ethanol stress, which was consistent with its higher cell growth and faster glucose consumption under the ethanol stress condition. We speculate that the ethanol tolerance of mutant strain may be due to some stress response mechanism. In the mutant strain, the expression level of some stress-responsive genes may be increased after exposure to ethanol. Therefore, further studies including transcriptomics and metabolomics are required to clarify its tolerance mechanism of RpoD mutation in conferring improved ethanol tolerance in *Z. mobilis*.

### Effects of ethanol stress on enzymatic activities

**Fig. 4:**
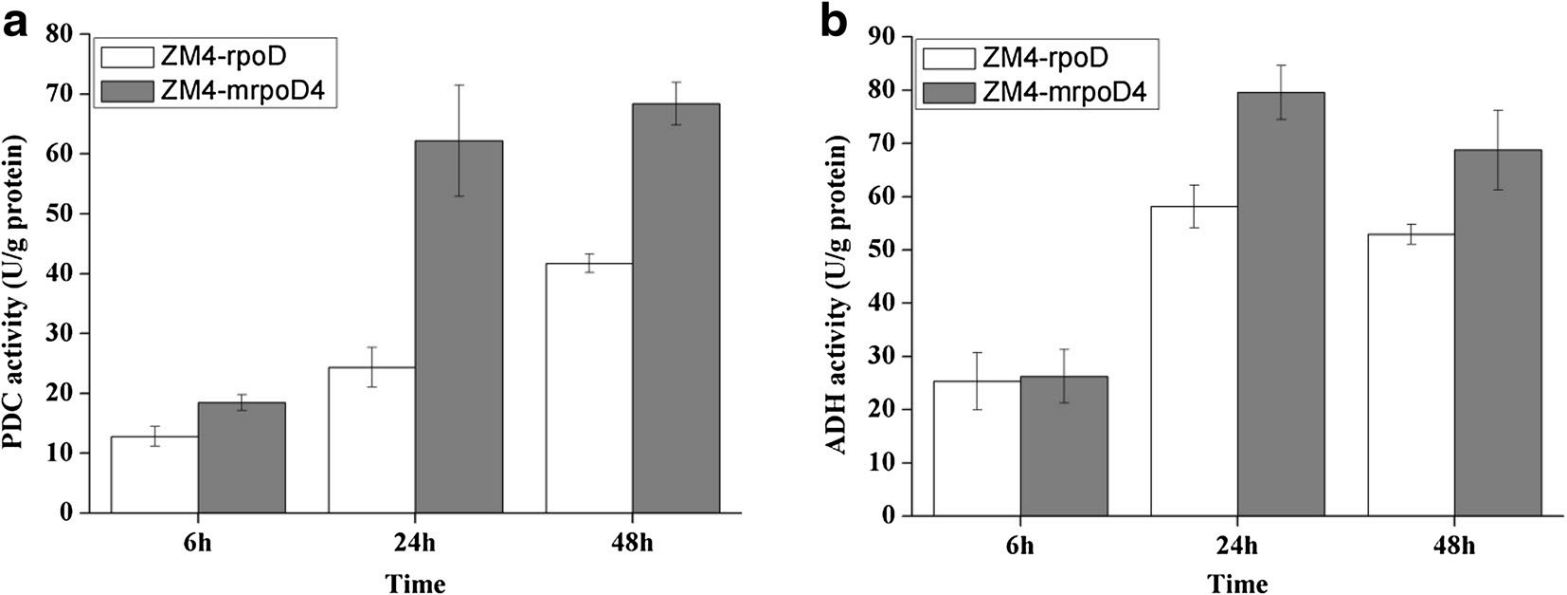
Pyruvate decarboxylase (PDC) and alcohol dehydrogenase (ADH) activities of crude extracts of ZM4-mrpoD4 and control strain under ethanol stress condition

For normal physiological operation of the Entner–Doudoroff (ED) pathway, both PDC and ADH key enzymes are required in *Z. mobilis*. Given this importance, the PDC and ADH activities of ZM4-mrpoD4 and the corresponding control strain were compared under the initial ethanol (9 %) stress after incubations lasting 6, 24, and 48 h. The PDC and ADH activities of ZM4-mrpoD4 and the control strain were not significantly different at 6 h, while the activities of the two enzymes from ZM4-mrpoD4 were significantly higher than those of the control strain at both 24 h and 48 h (Fig. 4). To this end, the PDC activity of ZM4-mrpoD4 was 62.23 and 68.42 U/g at 24 and 48 h under the initial ethanol (9 %) stress, respectively. These results show an increase of 2.6 and 1.6 times over that of the control strain. Similarly, the ADH activity of ZM4-mrpoD4 was also enhanced under conditions of the initial ethanol (9 %) stress, revealing increases of 1.4 and 1.3 times as that of control strain at 24 and 48 h.

**Fig. 5:**
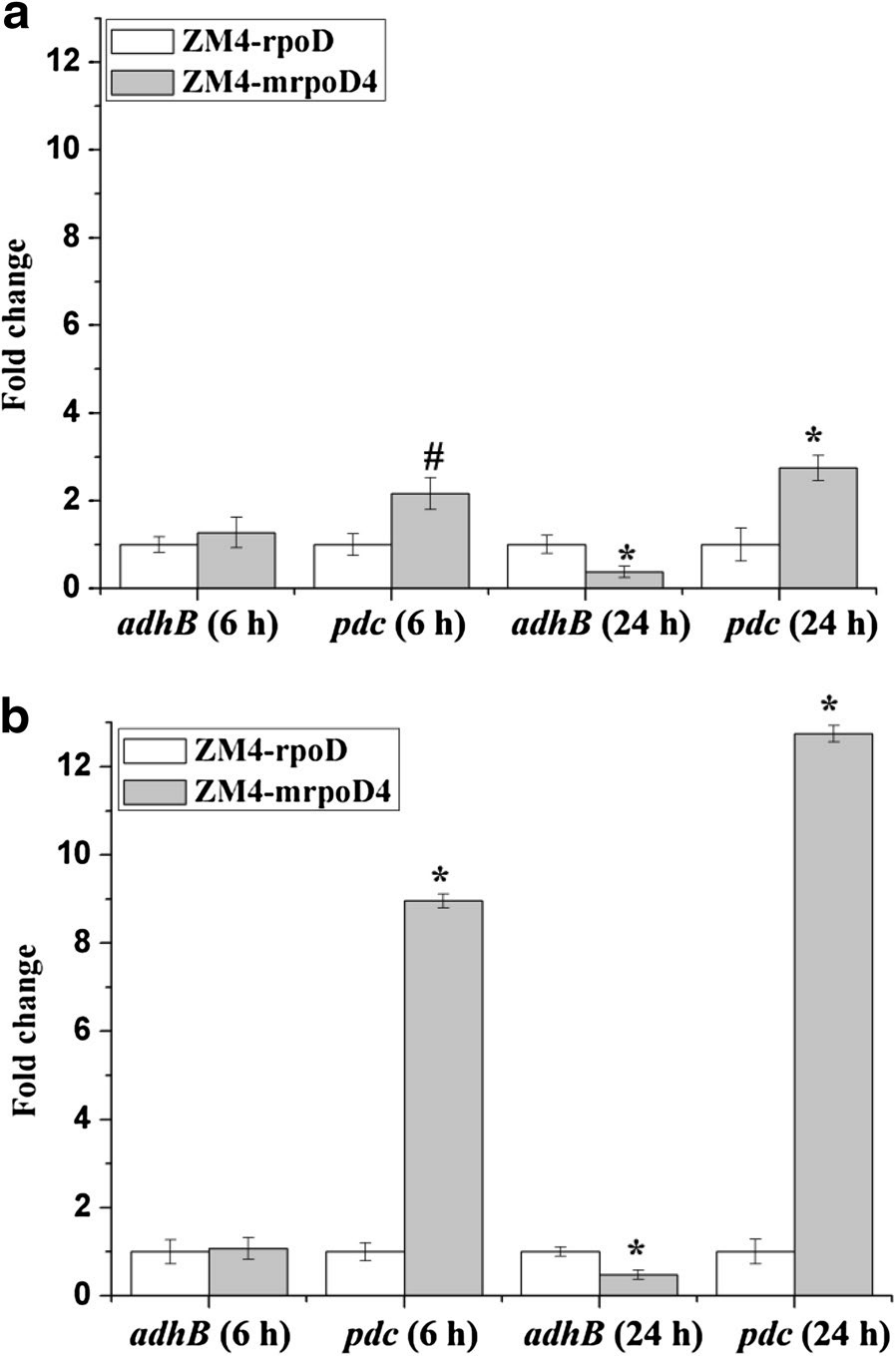
Fold changes in *adh*B and *pdc* gene expression levels of ZM4-mrpoD4 and control strains under different conditions. **a** no ethanol stress; **b** 9 % ethanol stress. ^#^ *p* < 0.05; *p < 0.01, compared with control strain using *t* test (mean ± SE, *n* = 3)

In addition, we measured transcription levels of *adhB* and *pdc* through the use of quantitative RT-PCR. In the absence of ethanol stress, our results showed that the expression level of the *adhB* gene in ZM4-mrpoD4 cultured for 6 h did not show differential expression from control strain. However, the *pdc* gene was up-regulated by about 2.2-fold (*p* > 0.05). The expression levels of the *adhB* and *pdc* genes in a 24 h ZM4-mrpoD4 culture were down- and up-regulated by about 0.6- and 2.7-fold (*p* > 0.01), respectively (Fig. 5). When the cells were exposed to the initial ethanol (9 %) stress for 6 h, the expression level of *adhB* in ZM4-mrpoD4 was not statistically different from that of control strain. In contrast, ZM4-mrpoD4 cultured for 24 h had *adhB* levels down-regulated by about 0.5-fold (*p* > 0.01). Interestingly, the level of *pdc* mRNA in ZM4-mrpoD4 cultured for either 6 or 24 h increased by 9.0- and 12.7-fold, respectively, when compared to the control strain (*p* > 0.01)(Fig. 5). We should note that in our earlier global profiling study using 5 % ethanol stress, the expression levels of *pdc* and *adhB* were down-regulated by about 0.8- and 0.9-fold, respectively [16].

### Sequence alignment and mutational analysis of the mutants

**Fig. 6:**
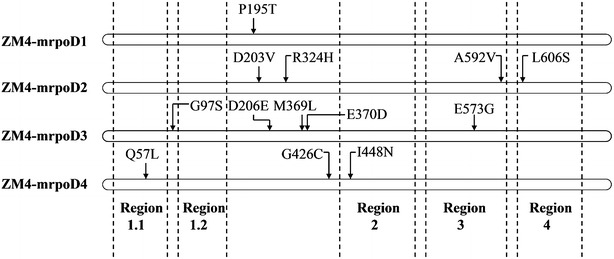
Summary of the mutation sites found in the primary sequence of the four ethanol tolerant mutants

Mutant *rpoD* genes were also sequenced using the primers 1623 *Xho*I F and 1623 *Xba*I R (Table 1). Sequences were aligned and compared using Clustal W version 2.0. Their amino acid substitutions are summarized in Table 2 and Fig. 6. As shown in Table 2 and Fig. 6, 13 total point mutations (Q57L, G97S, P195T, D203V, D206E, R324H, M369L, E370D, G426C, I448N, E573G, A592V and L606S) were adopted by these RpoD mutants to cope with the ethanol stress. Among them, three point mutations (R324H, M369L and E370D) are located at non-essential regions, making their function unknown, but allowing for their removal without a corresponding loss of function. Two point mutations (E573G and A592V) fell into conserved region 3, and three substitutions (Q57L, I448N, and L606S) were present in regions 1.1, 2, and 4, respectively. Our current hypothesis is that these mutations exert differential effects on promoter recognition and transcription initiation.

**Table 2:** Amino acid substitutions in four mutant strains

Mutants	Amino acid substitution
ZM4-mrpoD1	P195T
ZM4-mrpoD2	D203V R324H A592V L606S
ZM4-mrpoD3	G97S D206E M369L E370D E573G
ZM4-mrpoD4	Q57L G426C I448N

RpoD is an RNA polymerase sigma subunit composed of N-terminal domain of region 1.1 (residues 18–88) and region 1.2 (residues 116–149), non-essential region (residues 245–405) and C-terminal domain of region 2 (residues 437–507), region 3 (residues 516–593) and region 4 (residues 599–657) (NCBI conserved domain 2015) (Fig. 6). Analysis of the mutations found in the four mutants revealed several interesting features. First, we found that simple modification to the sigma factor RpoD led to enhanced strain tolerance towards ethanol stress. Second, the mutations occurred in all four of the predicted conserved regions, with the exception of mutant ZM4-mrpoD1, whereby only one mutation was found in P195T and did not include in any of the conserved regions (Fig. 6). Furthermore, though some of mutations are located in the conserved regions of the protein, none of the DNA binding residues (T610, R620, T629, L630, T641, E643, R644, R646, Q647, I648, A650, K651 and L653) on conserved domain region 4 were mutated.

The mutant ZM4-mrpoD4 with the highest ethanol tolerance had three mutations present (Q57L, G426C and I448N). Residue Q57 is one of the residues of region 1.1, which is known to be responsible for modulating DNA and promoter binding to allow for proper transcription initiation [35]. Given this role, it is possibly that mutation Q57L could influence DNA and promoter binding to RNA polymerase. Residue I448 lies in region 2, which contains both the −10 promoter recognition helix and the primary core RNA polymerase binding determinant [35]. It is therefore possible that point mutation I448N may also impact transcription through currently unknown mechanism. However, the structure–function relationship between these mutations and the observed ethanol tolerances remains unclear and in need to future investigation. To this end, further studies are required to identify its direct target genes and/or interacting partners to better elucidate the molecular mechanisms behind how mutations in RpoD can confer improved ethanol stress tolerance in *Z. mobilis*. Moreover, it will be interesting to ascertain the global transcriptional differences in strains harboring the mutation to ultimately identify the gene expression changes resulting in enhanced ethanol tolerance.

Having the sequenced genome of *Z. mobilis* ZM4 allows for better efforts at strain development [36]. In our previous study, our lab successfully used microarray technology to investigate expression profiling of the ethanologenic *Z. mobilis* ZM4 in response to ethanol stress [16]. Our results showed 127 genes were either up- or down-regulated in response to ethanol stress. Among these, sigma factors—those responsible for stress tolerance in *E. coli*—were also shown to be highly differential in their expression. These included sigma-E (*σ*^*E*^, ZMO1404, 1.3-fold), *σ*^*70*^ (*rpoD*, ZMO1623, 1.7-fold), *σ*^*54*^ (*rpo*N, ZMO0274, 1.2-fold), and *σ*^*28*^ (*fli*A, ZMO0626, 1.4-fold). Seo et al. [36] supposed that sigma-E plays a key role in resisting high ethanol condition in *Z. mobilis*, which is in keeping with our current results . In further support, Palonen et al. [37] also suggested that sigma-E is significantly involved in the stress tolerance of *Yersinia pseudotuberculosis* IP32953 . In the present study, RpoD mutation augmented *Z. mobilis* ethanol tolerance. Our results suggest that sigma 70 may also play an important role in resisting high ethanol concentration in *Z. mobilis*, with manipulation of σ^70^ allowing for another avenue for strain improvement.

## Conclusions

The present study used global transcriptional engineering tools to enhance the ethanol tolerance of *Z. mobilis* by rewiring its global regulator, RpoD. Mutations were introduced into RpoD via error-prone PCR and an enrichment screening procedure to isolate RpoD variants with enhanced ethanol resistance. Four mutants with enhanced ethanol tolerance were identified from error-prone PCR libraries. All mutants exhibited much better tolerance towards ethanol stress. Both the best ethanol-tolerant strain ZM4-mrpoD4 and its rebuilt mutant strain ZM4-imrpoD consumed glucose faster and produced more ethanol under ethanol stress conditions when compared to the control strain. Methodologically, our results further suggest that global transcription machinery engineering (gTME) is a viable route for strain engineering aimed at improving the complex phenotypes in *Z. mobilis*.

## Abbreviations

ED: Entner–Doudoroff
PDC: pyruvate decarboxylase
ADH: alcohol dehydrogenase
gTME: global transcription machinery engineering
HPLC: high performance liquid chromatography
